# Artificial Neural Network Modeling and Genetic Algorithm Optimization for Cadmium Removal from Aqueous Solutions by Reduced Graphene Oxide-Supported Nanoscale Zero-Valent Iron (nZVI/rGO) Composites

**DOI:** 10.3390/ma10050544

**Published:** 2017-05-17

**Authors:** Mingyi Fan, Tongjun Li, Jiwei Hu, Rensheng Cao, Xionghui Wei, Xuedan Shi, Wenqian Ruan

**Affiliations:** 1 Guizhou Provincial Key Laboratory for Information Systems of Mountainous Areas and Protection of Ecological Environment, Guizhou Normal University, Guiyang 550001, China; fanmingyifmy@163.com (M.F.); tongjunxiaoyu@163.com (T.L.); 18230825324@163.com (R.C.); xuedanshi1991@163.com (X.S.); wenqianruan@yahoo.com (W.R.); 2 Department of Applied Chemistry, College of Chemistry and Molecular Engineering, Peking University, Beijing 100871, China; xhwei@pku.edu.cn

**Keywords:** nZVI/rGO composites, Cd(II), removal, artificial neural network, genetic algorithm

## Abstract

Reduced graphene oxide-supported nanoscale zero-valent iron (nZVI/rGO) composites were synthesized in the present study by chemical deposition method and were then characterized by various methods, such as Fourier-transform infrared spectroscopy (FTIR) and X-ray photoelectron spectroscopy (XPS). The nZVI/rGO composites prepared were utilized for Cd(II) removal from aqueous solutions in batch mode at different initial Cd(II) concentrations, initial pH values, contact times, and operating temperatures. Response surface methodology (RSM) and artificial neural network hybridized with genetic algorithm (ANN-GA) were used for modeling the removal efficiency of Cd(II) and optimizing the four removal process variables. The average values of prediction errors for the RSM and ANN-GA models were 6.47% and 1.08%. Although both models were proven to be reliable in terms of predicting the removal efficiency of Cd(II), the ANN-GA model was found to be more accurate than the RSM model. In addition, experimental data were fitted to the Langmuir, Freundlich, and Dubinin-Radushkevich (D-R) isotherms. It was found that the Cd(II) adsorption was best fitted to the Langmuir isotherm. Examination on thermodynamic parameters revealed that the removal process was spontaneous and exothermic in nature. Furthermore, the pseudo-second-order model can better describe the kinetics of Cd(II) removal with a good R^2^ value than the pseudo-first-order model.

## 1. Introduction

Heavy metals, being controversial in their definition, often refer to elements having atomic weights between 63.5 and 200.6, and a specific gravity greater than 5.0 [1]. Heavy metal pollution has been considered as a serious environmental problem during the past few decades [2]. Contamination of the ecological environment with heavy metals may occur via both natural and anthropogenic causes [3]. Among all heavy metals, cadmium (Cd) is regarded as one of the most toxic because of its high mobility and persistence, which can accumulate easily and can pose dangers to human health [4,5,6]. Exposure to Cd may cause damage to human organs, such as the kidneys, livers, lungs, immune and reproductive systems [7]. Cd(II) is commonly present in industrial effluents from the manufacturing of cadmium–nickel batteries, fertilizers, pesticides, pigments, and dyes [8,9,10]. Therefore, the removal of Cd(II) from industrial effluents has an important significance to the human society [11].

Various methods have for several decades evolved in wastewater treatment, such as adsorption, electrochemical treatment, oxidation, ozonation, photochemical treatment, and froth flotation. Adsorption techniques have been widely investigated for the removal of heavy metals from wastewaters in recent years and a variety of *Nanomaterials* has hitherto been reported to serve as adsorbents for wastewater decontamination [12,13,14]. The versatility of nanoscale zero-valent iron (nZVI) has been considered a promising material utilized for the remediation of wastewater because of its small particle size, large specific surface area, and high reactive activity [15,16]. However, it still has some technical limitations, such as shortage of durability and mechanical strength, and poor stability, which may hinder the practical application of nZVI particles [17,18]. In addition, the aggregation is difficult to be avoided because of the magnetic interaction among the nZVI particles [19].

In order to overcome these shortcomings, methods have been developed using some materials as mechanical supports to strengthen the dispersion of the nZVI particles. Graphene as a new synthetic 2D allotrope of carbon has advantages as a supporter because of its high surface area and chemical stability. On the one hand, graphene oxide (GO) has many oxygen-containing functional groups on its basal plane and on the edges of its sheets, which can effectively combine the heavy metals and organic pollutants [20]. Yang et al. indicated that the GO has eminent adsorption capacity (46.6 mg/g) for Cu(II), which was around 10 times that of active carbon [21]. Gao et al. also found that the GO is a potential adsorbent for tetracycline antibiotics (313 mg/g) [22]. On the other hand, the graphene based composites have been exhibited to be effective in wastewater treatment with a higher efficiency than either of their pure components [23,24,25,26]. Compared with the nZVI particles, the lead removal efficiency of the nZVI/rGO composites increased by 25% [25].

As generally known, the performance of a removal process is a function of solution chemistry (initial pH, initial concentration, operating temperature, contact time, and other coexisting substances) and adsorbent structure [14]. The traditional method to model the removal process is originated from deep understanding of adsorption mechanisms. Mechanistic models (surface complexation model, MUSIC model, etc.) were adopted to manage these problems [27,28]. During the development of mechanistic models, the unavoidable simplifications and assumptions would offset the lack of knowledge of some unrevealed mechanisms (such as adsorption, reduction, and coprecipitation). However, it is unfortunate that these models could lead to inaccurate predictions. Statistical and mathematical methods (response surface methodology (RSM) and artificial neural network-genetic algorithm (ANN-GA)) are commonly employed to determine the optimal reaction conditions since they do not require mathematical description of the complex nature of the underlying process [29,30]. RSM is a collection of statistical techniques for designing experiments, building models, evaluating the effects of factors, and searching for the optimum conditions [31]. The most extensive application of RSM can be found in the industrial world in situations where a number of input variables affect some performance measures (responses), which are not easy or feasible to depict with a rigorous mathematical formulation [32]. A wide range of successful applications of RSM suggests that the second-order relation can reasonably approximate many of the wastewater treatment processes [33,34,35]. RSM has more frequently been employed than ANN, although both methods have been proven to be powerful tools for modeling and optimizing the wastewater treatment.

ANN was inspired by biological neurons and was derived from artificial intelligence (AI) research, which can effectively describe multivariate nonlinear problems with the suitable amount of data and the appropriate training algorithm applied [14]. ANN is a non-linear statistical data modeling in machine learning due to its ability to learn rather complicated functions. The main advantage of ANN over RSM is that ANN can approximate almost all kinds of the non-linear functions, whereas RSM is useful only for the quadratic approximations. The utilization of ANN can predict the degree of non-linearity without extensive experimentation as compared to that of the traditional mathematical models [36]. However, ANN cannot solve all problems in wastewater treatment and it has some limitations, i.e., it does not guarantee the global optimal solution [37]. Genetic algorithm is a numerical search tool which operates according to procedures that resemble the principles of natural selection and genetics. Previous studies took advantage of the genetic algorithm coupled with ANN (ANN-GA) to generate the global optimum operating variables for the studied processes [38,39,40].

In the present work, RSM and ANN-GA were employed to model and optimize the Cd(II) removal from aqueous solutions by the nZVI/rGO composites. The nZVI/rGO composites were prepared by chemical deposition method and were characterized by Fourier-transform infrared spectroscopy (FTIR) and X-ray photoelectron spectroscopy (XPS). Initial concentration, initial pH, contact time, and operating temperature were chosen as the process parameters for Cd(II) removal by RSM and ANN-GA. The relative importance of the independent variables was explored using analysis of variance and sensitivity analysis. The developed RSM model and the ANN-GA model were compared against each other with respect to the accuracy of Cd(II) removal efficiency prediction. The isotherms (Langmuir, Freundlish, and Dubinin–Radushkevich), thermodynamic (Van’t Hoff equation) and kinetic studies (pseudo-first-order, pseudo-second-order, and intraparticle diffusion equations) were preformed to explore the adsorptive behavior of Cd(II) onto the nZVI/rGO composites. In addition, the mechanisms for the removal of Cd(II) ions from aqueous solutions by the nZVI/rGO composites were investigated based on the XPS analysis.

## 2. Materials and Methods

### 2.1. Materials

All chemicals used in this study are of analytical grade from Sinopharm Chemical Reagent (Beijing, China) without further purification. The stock solution (1000 mg/L) of Cd(II) was prepared by dissolving CdCl_2_ in deionized water. Required initial concentration of Cd(II) in samples (5 mg/L to 300 mg/L) was achieved by appropriate dilution of the stock solution.

### 2.2. Preparation of nZVI/rGO Composites

The synthesis of graphene oxide (GO) was carried out by the modified Hummers method, and recduced graphene oxide (rGO) was synthesized by the reduction of GO with the NaBH_4_ solution [24,25]. 1 g of GO was dispersed in deionized water with ultrasonication for 2 h. 10 g of FeSO_4_ 7H_2_O was added to the GO suspension, which was mixed for 12 h with magnetic stirring to reach the adsorption equilibrium. Then, 50 mL of 0.14 mol/L NaBH_4_ solution was added into the above solution at room temperature with magnetic stirring for 30 min. The nZVI/rGO composites were separated by centrifugation and dried at 50 °C in the vacuum drying oven for 24 h [24].

### 2.3. Characterization of nZVI/rGO Composites

Fourier Transform infrared spectroscopy (FTIR) measurement of nZVI/rGO, GO, and rGO used a Nicolet 6700 spectrometer (Bruker, Berlin, Germany). The samples for the spectral analysis were prepared by mixing sample and KBr in 1:100 proportion, which were pressed into a sheer slice. Then, the average over six scans were collected for each measurement with a resolution of 4 cm^−1^, and the scan range was from 4000 to 400 cm^−1^. XPS analysis for the nZVI/rGO-Cd(II) composites was performed by using a hemispherical energy analyzer Phoibos 100/150 (SPECS, Berlin, Germany) in comparison with the XPS analysis of nZVI/rGO composites in our earlier work [24]. The other characterizations of nZVI/rGO composites—such as scanning electron microscopy, X-ray diffractometer, BET surface analyzer, and Raman spectroscopy—were carried out as mentioned in our earlier work [24].

### 2.4. Batch Experimental Program

Initial Cd(II) concentration (*X*_1_), initial pH (*X*_2_), contact time (*X*_3_), and operating temperature (*X*_4_) were considered as independent variables, and the removal percentage of Cd(II) ions (*Y*) was selected as the dependent variable. Batch removal experiments (single factor experiments) were carried out to study the effect of initial Cd(II) concentration (5–50 mg/L), initial pH (3–7), contact time (0–60 min), and operating temperature (20–50 °C) on the removal efficiency of Cd(II) in 100 mL centrifugal tubes. In batch experiments, the initial pH of Cd(II) solution was adjusted to the desired value by using 0.1 mol/L HCl or 0.1 mol/L NaOH. Specific amounts of nZVI/rGO composites (30 mg) were added into 50 mL solutions containing desired initial concentration. Then, the above solutions were shaken on temperature controlled water bath shaker (200 rpm) at required operating temperature and for different contact times. The nZVI/rGO composites were separated from aqueous solutions by a magnet after the removal experiments. The Cd(II) concentration in the sample solutions was determined through flame atomic absorption spectrophotometer (AAS), which was made by Ray Leigh Corporation, Beijing, China. All experiments were performed in triplicates and the average value of the obtained data was adopted for the analysis [41]. Equations for the calculation of different removal process parameters can also be referred to our earlier work [24].

### 2.5. Modeling and Optimization

#### 2.5.1. RSM Modeling and Optimization

RSM is a commonly used statistical method for optimizing the process parameters and building quadratic models providing the statistical relationship among the variables. The four independent variables—viz. initial Cd(II) concentration, initial pH, contact time, and operating temperature—were selected for designing the experiments by employing Box-Behnken Design (BBD) of RSM. The ranges of independent variables chosen are collected in Table 1, based on the single factor experiments (Figure S1). The data from BBD were subjected to a second-order multiple regression analysis and analysis of variance (ANOVA) to interpret the behavior of the system using the least squares regression methodology. The quadratic model can be represented as

(1) $$Y=c_{0}+\sum_{i=1}^{m}c_{i}x_{i}+\sum_{i=1}^{m}\sum_{j=1}^{m}c_{ij}x_{i}x_{j}+\sum_{i=1}^{m}c_{ii}{x_{i}}^{2}+a$$

where *Y* is the response (removal efficiencies of Cd(II) ions), *c*_0_ represents the constant coefficient, *c_i_*, *c_ii_* and *c_ij_* are the coefficient for linear, quadratic and interaction effect, respectively, *x_i_* and *x_j_* are the values of the independent variables; *a* represents the residual error.

#### 2.5.2. ANN-GA Modeling and Optimization

ANN is a superior and accurate modeling technique as compared to RSM because it represents the nonlinear relationships including quadratic equations in a better way [42]. Thus, a feed-forward multilayer perception (MLP) ANN model was employed with a back propagation (BP) algorithm to build the predictive mathematical model with scaled condition of the four inputs (e.g., initial Cd(II) concentration, initial pH, contact time, and operating temperature) and one output as Cd(II) removal efficiency. The neural network topology consisted of three layers: an input layer, a hidden layer, and an output layer. The first step in ANN was network training, which was done by utilizing 80% (=24 groups) experiments of BBD, while the performance of trained network was evaluated with rest 20% (=5 groups) BBD data. All input and output data were normalized between −1 and 1 to avoid numerical overflows due to large or small values [43]. Normalization of the input data can be computed as

(2) $$X_{i}=2\frac{X-X_{\min}}{X_{\max}-X_{\min}}-1$$

where *X_i_* is the normalized value of an input variable (*X*), *X*_min_ and *X*_max_ are the minimum and maximum values of variables, respectively. The optimized values of variables after completion of the training were rescaled to their real values according to the equation

(3) $$X=\frac{\left(X_{i}+1)(X_{\max}-X_{\min}\right)}{2}+X_{\min}$$

The network performance was computed in terms of mean squared error (MSE), which shows the statistical difference between experimental and predicted values. The MSE can be described as

(4) $$MSE=\frac{\sum_{i=1}^{i=M}{\left(y_{i,pred}-y_{i,\exp}\right)}^{2}}{M}$$

where *M* is the number of data sets, *y_i,pred_* represents the predicted data, and *y_i,exp_* is the experimental data.

The learning rate of the network was set to the values that resulted in an optimal coefficient of correlation (R^2^) for the neural network. The goodness of fit for the constructed model can also be represented in terms of R^2^ value, which is defined as

(5) $$R^{2}=\frac{\sum_{i=1}^{M}{\left(x_{i}-x_{mean}\right)}^{2}-\sum_{i=1}^{M}{\left(x_{i}-y_{i}\right)}^{2}}{\sum_{i=1}^{M}{\left(x_{i}-y_{i}\right)}^{2}}$$

where *x_i_* represents the target value for the *i*th data point, *x_mean_* stands for the mean target value, and *y_i_* is the output of the *i*th data point.

Determining the number of neurons in the hidden layer is an important task when an ANN is designed [44,45]. On the one hand, the convergence rate of the network may be affected by a few neurons in the hidden layer. On the other hand, a large number of neurons may result in the complicated network topology, training frequency increasing, model over-fitting, and generalization reduction. Therefore, the number of neurons in the hidden layer is obtained by trial and error method in order that the error between the experimental values and predicted values is minimized.

After an ANN model is developed with the good prediction accuracy and generalization ability, its input space (four independent variables) is optimized using genetic algorithm (GA). The optimization of GA can be depicted as a global optimization procedure with the advantage of being independent on the initial value to achieve the convergence. GA treats an optimization through a simple cycle of four stages, which consists of initialization of solution populations, fitness computation, selection of best chromosomes, genetic propagation of selected parent chromosomes using genetic operators like crossover and mutation. Both crossover and mutation are implemented to produce the new and better populations of chromosomes. The best operation condition that evolves after repeating the above cycle till convergence forms the optimization parameters to the problem [31].

### 2.6. Adsorption Isotherm Study

In order to establish the most suitable correlation for the equilibrium data in the design of adsorption system, three common isotherms were employed: the Langmuir, Freundlish, and Dubinin-Radushkevich (D-R) isotherms [46,47,48]. The Langmuir isotherm model is based on the supposition that the adsorption takes place on a homogenous surface by monolayer adsorption without the interaction between the adsorbate and the adsorbent. The general form of the Langmuir isotherm is

(6) $$\frac{C_{e}}{q_{e}}=\frac{1}{q_{m}k}+\frac{C_{e}}{q_{m}}$$

where *C_e_* (mg/L) is the equilibrium Cd(II) concentration, *q_e_* (mg/g) represents the amount of Cd(II) adsorbed per unit mass of adsorbent, *q_m_* (mg/g) stands for the maximum adsorption capacity depending on the number of adsorption sites, *k* (L/g) is the Langmuir constant related to the energy of adsorption.

The Freundlich isotherm assumes that the uptake of metal ions occurs on a heterogeneous surface by multilayer adsorption and that the amount of metal ions adsorbed increases infinitely with an increase in the concentration of metal ions. The general form of the Freundlich equation is given as

(7) $$\log q_{e}=\log K_{f}+\frac{1}{n}\log C_{e}$$

where *K_f_* and 1/n are the Freundlich constants depending on the temperature. The n is related to the adsorption energy distribution, and *K_f_* indicates the adsorption capacity. A relatively slight slope *n* < 1 indicates that sorption intensity is favorable over the entire range of concentration studied, while a steep slope (*n* > 1) means that sorption intensity is favorable at high concentrations but much less at lower concentrations [49]. 1/n in the Freundlich model is a measure of adsorption intensity or surface heterogeneity. The value of 1/n below 1 indicates a normal Freundlich isotherm, and the adsorption becomes more heterogeneous as the value of 1/n gets closer to zero [50].

The D-R isotherm is generally applied to describe the adsorption mechanism with a Gaussian energy distribution onto a heterogeneous surface [51]. The D-R sorption isotherm is more general than the Langmuir isotherm, as its derivation is not based on ideal assumptions, such as equipotent of the sorption sites, absence of steric hindrance between adsorbed and incoming particles and surface homogeneity on the microscopic level. The linear form of the D-R isotherm is given as

(8) $$\ln q_{e}=\ln q_{m}-\beta \varepsilon^{2}$$

where *β* represents the constant of the sorption energy (mol^2^/J^2^) related to adsorption mean free energy (kJ/mol), and *ε* is Polyanyi potential—*ε* is defined as

(9) $$\varepsilon =RT\ln(1+\frac{1}{C_{e}})$$

where *T* is the solution temperature (K) and *R* is the gas constant and is equal to 8.314 J/mol/K.

The adsorption mean free energy (*E*, kJ/mol), defined as the free energy change when one mol of ion in the solution is moved from infinity to the adsorbent surface, which can give information about physical or chemical adsorption mechanism [52]. This can be computed as

(10) $$E=\frac{1}{\sqrt{2\beta }}$$

If this value is below 8 kJ/mol, the adsorption is physical, and when it lies between 8 and 16 kJ/mol, the adsorption process takes place chemically [53].

### 2.7. Thermodynamic Study

The thermodynamic parameters are indispensable to evaluate the feasibility and nature of the removal process. Thus, isotherm data, related to adsorption of Cd(II) onto the nZVI/rGO composites at various temperatures ranging from 293 to 323 K, can be analyzed to obtain the value of thermodynamic parameters. The values of thermodynamic parameters (enthalpy change (Δ*H*) and entropy change (Δ*S*)) were calculated using the Van’t Hoff equation

(11) $$\ln k_{c}=\frac{\Delta S}{R}-\frac{\Delta H}{RT}$$

where *k_c_* is the constant of Langmuir (L/mol), *R* is the gas constant (8.314 J/mol/K), *T* is the temperature (K), Δ*S* denotes the standard entropy change (J/mol/K), and Δ*H* is the standard enthalpy (J/mol). The values of Δ*G* can be calculated by the Gibbs–Helmholtz

(12) ΔG=ΔH−TΔS

### 2.8. Removal Kinetics

In order to evaluate the efficiency of the studied nZVI/rGO composites for removing Cd(II) ions, kinetic data were modeled by the pseudo-first-order equation, pseudo-second-order equation, and intraparticle diffusion equation [54]. These equations are given as

(13) $$\log(q_{e}-q_{t})=\log q_{e}-k_{1}t/2.303$$

(14) $$t/q_{t}=1/k_{2}{q^{2}}_{e}+t/q_{e}$$

(15) $$q_{t}=k_{3}t^{1/2}+b$$

where *q_e_* and *q_t_* are the sorption capacity (mg/g) at equilibrium time and at time *t*, respectively; *k*_1_, *k*_2_, and *k*_3_ are the pseudo-first-order rate constant (1/min), pseudo-second-order rate constant (g/mg/min), and intraparticle diffusion rate constant (mg/g/min^1/2^), respectively; *b* (mg/g) is a constant of intraparticle diffusion model.

## 3. Results and Discussion

### 3.1. Characterization of nZVI/rGO Composites

**Figure 1:**
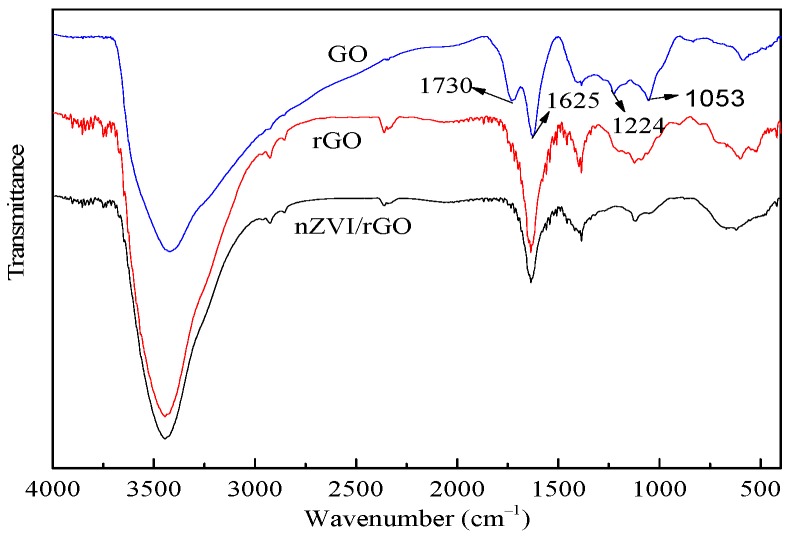
FTIR spectra of GO, rGO, and nZVI/rGO.

Figure 1 shows the FTIR spectra of GO, rGO, and nZVI/rGO. The FTIR spectrum of the GO consists of C=O (1730 cm^−1^), aromatic C=C (1625 cm^−1^), epoxy C–O (1224 cm^−1^) and alkoxy C–O (1053 cm^−1^) stretching vibrations. The FTIR spectra of the rGO and nZVI/rGO consisted of an apparent peak at 1625 cm^−1^ corresponding to the aromatic C=C stretch. However, the intensity of the C=O peak at 1730 cm^−1^ was significantly reduced, which demonstrated that the most of the oxygen–containing functional groups were removed. The FTIR spectrum of the nZVI/rGO composites is similar to that of rGO indicating the presence of rGO in the nZVI/rGO composites, which also indicated that the GO was fully reduced to rGO.

### 3.2. RSM Optimization

Table 2 shows the values of the dependent and independent variables and the observed and predicted experimental data for the removal of Cd(II). As shown in Table 2, the removal efficiency of Cd(II) increased from 44.26% to 77.25%. The center point (0, 0, 0, 0) was repeated for five times and nearly the same results obtained showed the reproducibility of the data. The quadratic model was generated by the RSM design, which was applied to evaluate the responses. The developed RSM model equation is given as follows:

*Y* = − 98.09 + 2.67*X*_1_ + 40.93*X*_2_ − 0.08*X*_3_ − 0.89*X*_4_ − 0.36*X*_1_*X*_2_ − 0.03*X*_1_*X*_3_ + 0.03*X*_1_*X*_4_ + 0.29*X*_2_*X*_3_ + 0.36*X*_2_*X*_4_ + 0.03*X*_3_*X*_4_ − 0.03*X*_1_^2^ − 3.33*X*_2_^2^ − 0.02*X*_3_^2^ − 0.03*X*_4_^2^ (16)

The result of ANOVA for the second-order model is shown in Table 3, which indicated that this model was significant with a low probability value with F value of 113.96. Statistical analysis of the data showed the values of 0.9913 and 0.9826 for R^2^ and adjusted R^2^, which demonstrated that only 0.87% of the total variation could not be interpreted by the quadratic model. In addition, the initial Cd(II) concentration, initial pH, contact time and operating temperature are quite significant for the Cd(II) removal. Judging by *F*-values of independent variables, the decrease order for these parameters influencing the removal process is: initial Cd(II) concentration > initial pH > operating temperature > contact time. The predicted maximum for the removal of Cd(II) by using RSM was 85.92%, and the optimized conditions were *X*_1_ = 20.00 mg/L, *X*_2_ = 7.00, *X*_3_ = 30.00 min, and *X*_4_ = 37.13 °C. The Cd(II) removal obtained experimentally at RSM optimized condition was 80.36 ± 0.46% with 6.47% of the prediction error.

### 3.3. ANN-GA Optimization

**Figure 2:**
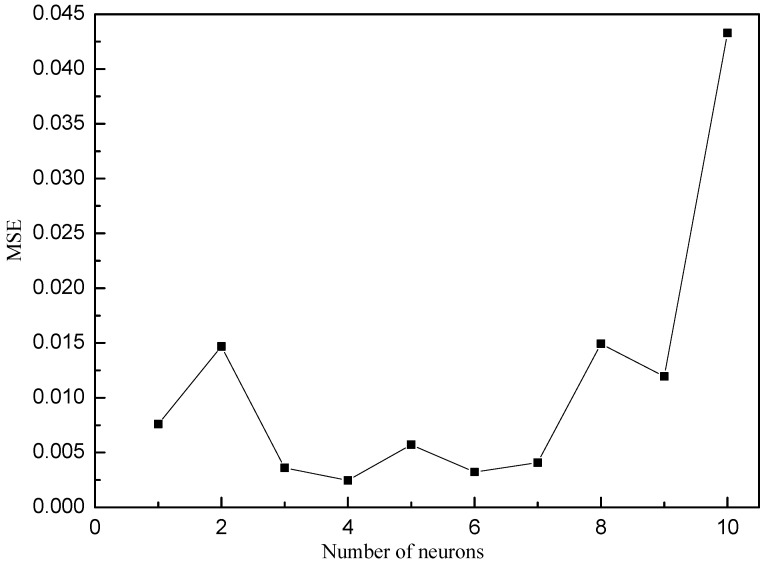
Effect of neuron number in the hidden layer on the performance of BP-ANN using MSE.

**Figure 3:**
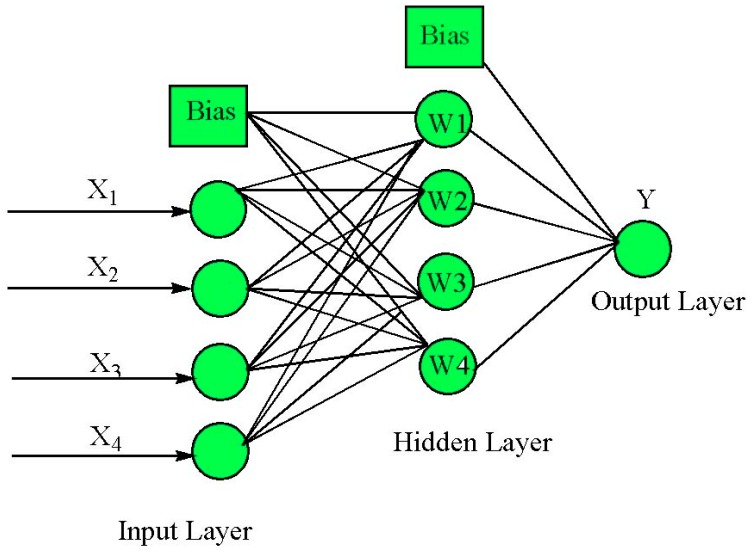
Pattern of optimized ANN architecture (4-4-1).

The optimum number of neurons was determined based on the minimum value of MSE for the training and test set [12]. The optimization was done by using the variable learning rate back-propagation (traingdx) as a training algorithm with the neuron number in the range from 1 to 10. MSE was 0.007595 when one neuron was used and decreased to 0.002461 when four neurons were selected (Figure 2). Figure 3 shows the optimized neural network structure (4-4-1), which has the tangent sigmoid transfer function (tansig) at hidden layer with four neurons and linear transfer function (purelin) at output layer.

**Figure 4:**
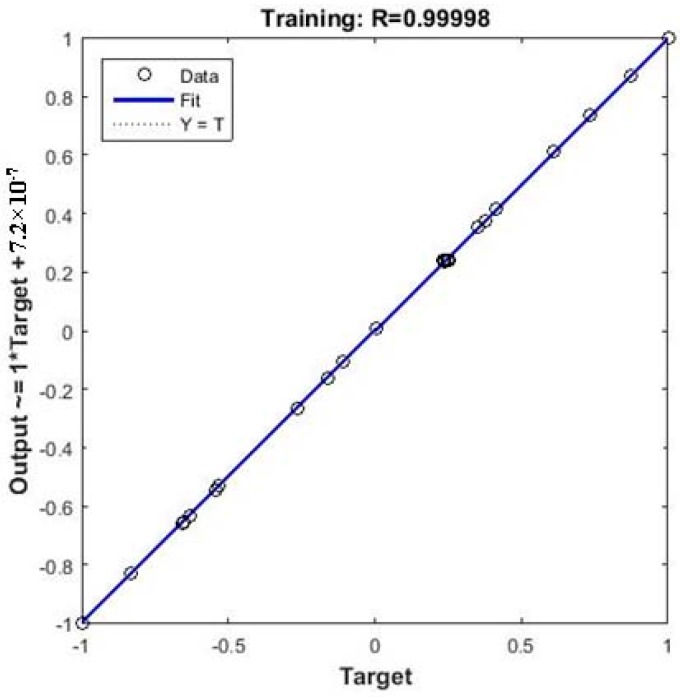
Correlations between data predicted by BP-ANN model and experimental data.

**Figure 5:**
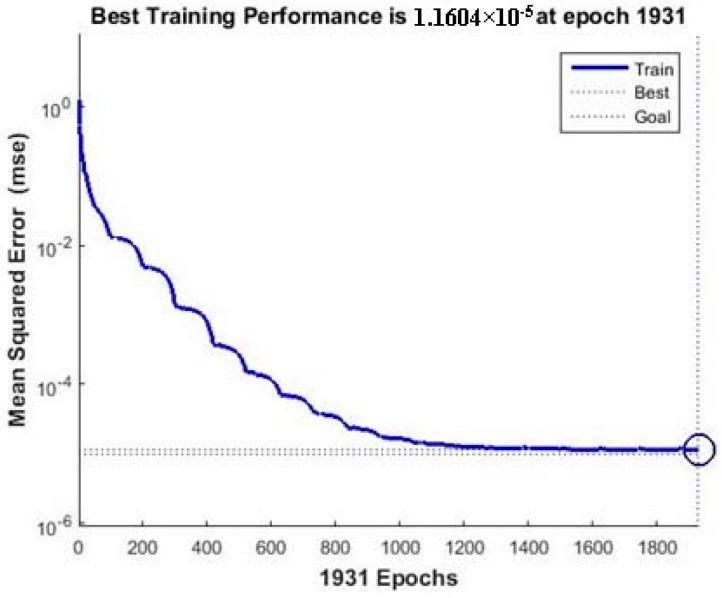
The performance of the BP-ANN model (4-4-1) in the training phase showing performance value (MSE-0.0000116) after 1931 epochs.

Figure 4 shows a comparison between experimental Cd(II) removal values and predicted values using the BP-ANN model. The linear fit was indicated by a solid line with 0.9999 of the R^2^, which indicates a good agreement between the predicted data and experimental data. The MSE decreases rapidly in the beginning, and the reduction rate slows down with an rise in the number of epochs, finally reaching a saturation point when the model was trained for 1931 epochs (Figure 5). The average errors (%) between the experimental and predicted Cd(II) removal efficiency for the training and test set were 0.0012 and 2.88, respectively. Small values of the MSE and high values of the R^2^ for both the training and test outputs demonstrated that the BP-ANN model possesses good approximation and generalization characteristics.

Sensitivity analysis was performed to sum the connection weights of the ANN model, which can make us understand the in-depth information of the ANN model. Connection weight is to evaluate the variable importance through the calculation that records the input-hidden and hidden-output connection weights between each independent variable and dependent variables across all hidden neurons [55]. The Garson equation is useful to exhibit the relative influence of the independent variables on the dependent variable, which can be described as

(17) $$P_{av}=\frac{\sum_{b=1}^{N}\left(\frac{|w_{ab}|}{\sum_{d=1}^{M}\left|w_{db}\right|}\left|e_{bv}\right|\right)}{\sum_{a=1}^{M}\left(\sum_{b=1}^{N}\left(\frac{\left|w_{ab}\right|}{\sum_{d=1}^{M}\left|w_{db}\right|}\left|e_{bv}\right|\right)\right)}$$

where *P* is the percentage of influence for the input neuron, *w* represents the weight between input and hidden neuron, *e* stands for the weight between hidden and output neuron, *M* is the number of neurons in input layer, *N* is the number of neurons in hidden layer, and *v* is the number of output neuron. As shown in Table 4, initial concentration (43.33%) and initial pH (30.71%) have a strong effect on the removal of Cd(II), which was consistent with the results of ANOVA.

**Figure 6:**
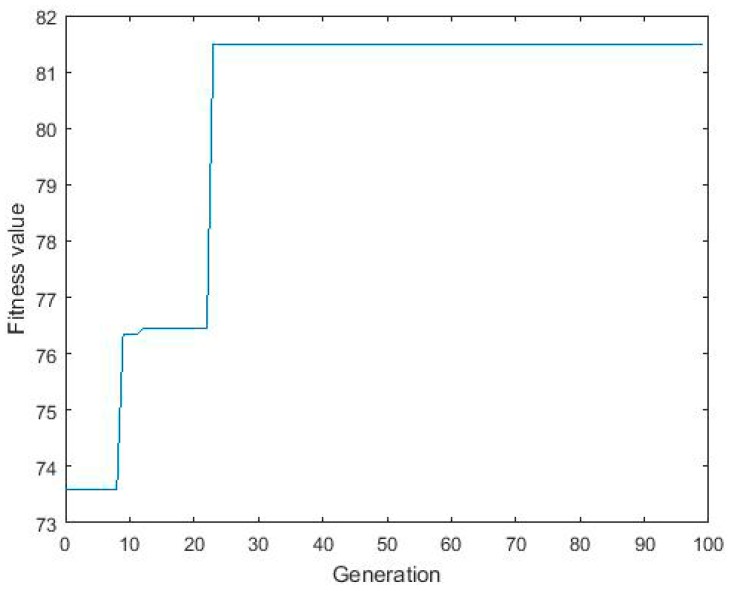
Evolvement of fitness with 100 generations.

In order to achieve an optimum solution, the process of optimization was iterated at variable GA input conditions. Achievement of similar results for most of the GA inputs ensured that it must be global optimal solution [56]. GA generated the optimal set of initial Cd(II) concentration, initial pH, contact time, and operating temperature that governed a maximum removal of Cd(II). The best fitness plot achieved during the iterations of GA after 23 generations describes the gradual convergence of results towards the optimal solution (Figure 6). The ANN predicted Cd(II) removal at the GA optimized condition was 81.50% at the condition of 20.16 mg/L (initial Cd(II) concentration), 6.48 (initial pH), 30.00 min (contact time), and 25.31 °C (operating temperature). This result was cross-validated by performing batch experiments at the GA-specified optimum condition. The removal of Cd(II) achieved during experimental condition was 82.38 ± 0.82%, which was in a good agreement with the ANN-GA prediction.

### 3.4. Comparison of RSM and Hybrid ANN-GA

The RSM model and the ANN-GA model were compared and validated by performing confirmatory experiments (in triplicate) at the predicted optimal operating condition. The deviation of the predicted results and the average values of prediction errors between the RSM model and the ANN-GA model are shown in Table 5. It was found that the average values of prediction errors of RSM and ANN-GA models were 6.47% and 1.08% and the R^2^ of ANN-GA (0.9999) was higher than that of RSM (0.9913). This difference between predicted and experimental removal efficiency can be contributed to the extent of deviation in predictive capacity of model. Since ANN is more accurate and more generalized model than quadratic RSM, it better obtained the global optimum.

Although ANN is superior to RSM, these models complement each other in interpreting the experimental removal efficiency. ANN is more reliable in capturing the nonlinear relationship between the removal efficiency and process variables, while RSM notes the statistical importance of the individual process variables and their interactions via ANOVA. However, the disadvantage of RSM is that it can only assume a quadratic non-linear correlation for the removal process. Therefore, the effectively used RSM can be achieved when the search space is appropriately narrowed down, which makes the modeling process highly dependent upon the search space. This will require either additional experiments or good prior knowledge of the removal process to fix the search space. ANN can easily overcome the above disadvantage of RSM since it can inherently capture almost any form of non-linearity. Hence, the liberal search space can be chosen by using ANN although the correlation in the search space is more complex than the quadratic.

### 3.5. Adsorption Isotherms

**Figure 7:**
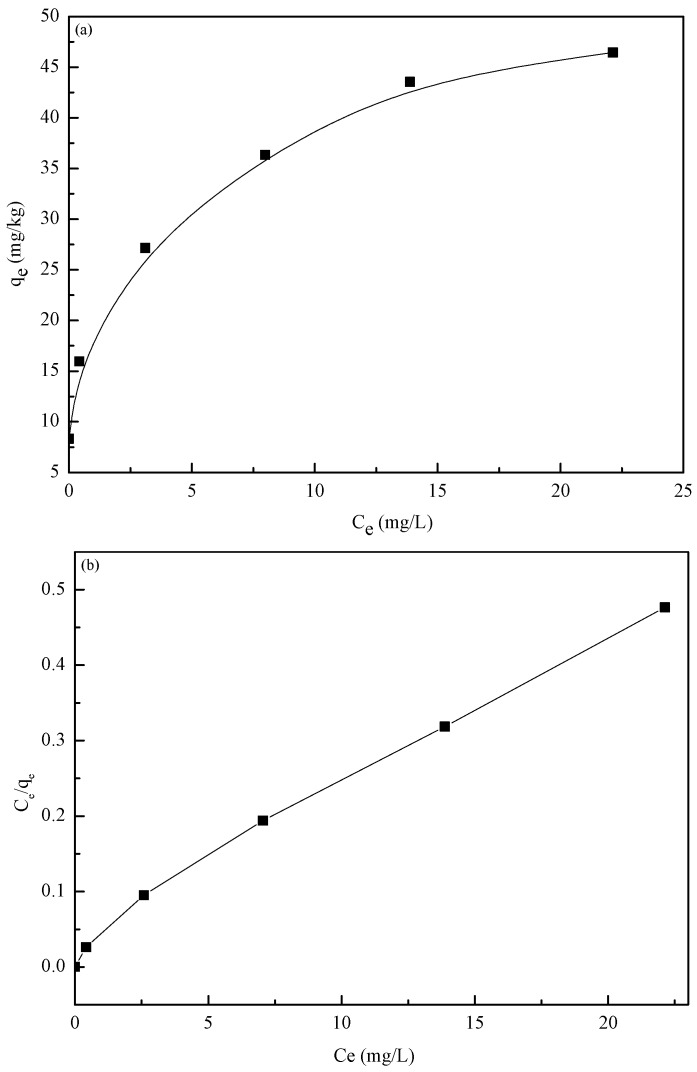
(**a**) Effect of initial concentration on the Cd(II) ions adsorption on nZVI/rGO composites; (**b**) the relationship between *C_e_*/*q_e_* and *C_e_*. (Initial pH = 7.0; nZVI/rGO composites dosage = 30 mg; operating temperature = 20 °C; and contact time = 1 h).

Equilibrium isotherm studies were carried out with different initial Cd(II) concentrations (5–50 mg/L) (Figure 7a). The quantity of Cd(II) adsorbed on the adsorbents increases with the rise in initial Cd(II) concentrations, since higher initial concentrations provide a higher driving force for the ion transportation from the solution to the adsorbents, resulting in an increase in collisions between Cd(II) and active sites on the adsorbents [18]. The linear relationship between *C_e_*/*q_e_* and C_e_ showed that the Langmuir isotherm fit well with the equilibrium data of Cd(II) ions by the nZVI/rGO composites (Figure 7b).

Table 6 shows the values of R^2^ and other parameters of the three models. As can be seen, the R^2^ values of the three models give the values higher than 0.97, however fitting the data using the Langmuir model gives a better fit than when using the Freundlich and D–R models. It can be seen from the fitting data obtained that the value of R^2^ is high for the Langmuir isotherm (0.9909) compared to that of the Freundlich isotherm (0.9852) and D–R isotherm (0.8226). In addition, the value of the parameter K_f_ is 20.02, which is higher than that reported by other studies [57,58,59,60]. This shows the remarkable Cd affinity of the nZVI/rGO composites compared to other adsorbents. The n value of the Freundlich isotherm was greater than 1, indicating that the adsorption intensity was favorable at high concentrations. The calculated value of 1/n was 0.28, which suggests the adsorption process was heterogeneous. Moreover, the value of E is found to be 2.24 kJ/mol, indicating that the adsorption of Cd(II) onto the nZVI/rGO composites is carried out through a physical interaction. Therefore, these results suggest that the adsorption process of Cd(II) onto the nZVI/rGO composites could take place by monolayer physical adsorption in nature.

On the basis of the Langmuir isotherm, the maximum adsorption value (*q_m_*) of the nZVI/rGO composites to Cd(II) is 47.84 mg/g, which is close to the experimental value (46.45 mg/g). Satisfactory fitting of the experimental data to the Langmuir isotherm may be attributed to the homogeneous distribution of active sites on the surface of the nZVI/rGO composites. The adsorption capacity of Cd(II) using the nZVI/rGO composites was lower than that of Pb(II) (904 mg/g) determined in our earlier work [24]. The reason for this is the physicochemical properties of Cd(II), which has the lower covalent index, atomic weight, electronegativity, electrode potential, and ionic size than Pb(II) [61,62].

A further analysis of the Langmuir equation can be made on the basis of dimensionless equilibrium parameters [63], *R_L_* also known as the separation factor, which was calculated with the equation

*R_L_* = 1/(1 + k*C*_0_) (18)

where *C*_0_ represents the initial Cd(II) concentration (mg/L) and *k* stands for the Langmuir constant indicating the nature of adsorption. The value of *R_L_* indicates the nature of the adsorption process to be irreversible (*R_L_* = 0), favorable (0 < *R_L_* < 1), linear (*R_L_* = 1), or unfavorable (*R_L_* > 1). The calculated *R_L_* values in our work for the adsorption on the nZVI/rGO composites at different initial Cd(II) concentrations are presented in Table 7. As can be seen here, the *R_L_* values obtained were all between 0 and 1, indicating that this adsorption process is favorable.

### 3.6. Thermodynamic Study

**Figure 8:**
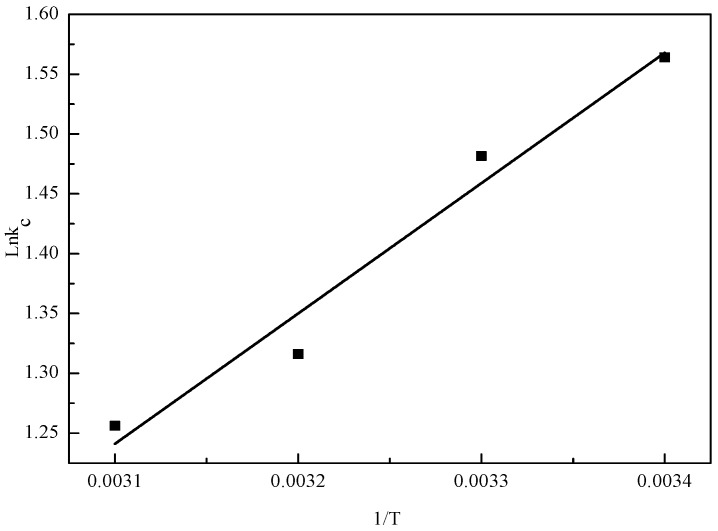
Van’s Hoff plot for the Cd(II) removal by the nZVI/rGO composites.

Van’t Hoff plots were drawn in the temperature range of 20–50 °C (Figure 8) and the thermodynamic parameters were calculated using thermodynamic equations (Table 8) [64,65]. The negative values of Δ*H* confirmed the exothermic nature of the removal process and negative magnitude of Δ*G* decreased from −25.7752 to −27.5363 kJ/mol, which signified the thermodynamically feasible and spontaneous nature of the adsorption process. It is reported that when the Δ*H* value is lower than 40 kJ/mol, the adsorption type can be described as a physical process [52]. ΔH in the present work was −8.5759 kJ/mol indicating that the adsorption process is a physical adsorption, which was in agreement with the result of D-R model. Furthermore, positive values of Δ*S* demonstrated the increase in degree of randomness of metal ions during removal process this might be due to the disorderliness at solid-liquid interface [66,67].

### 3.7. Removal Kinetics

**Figure 9:**
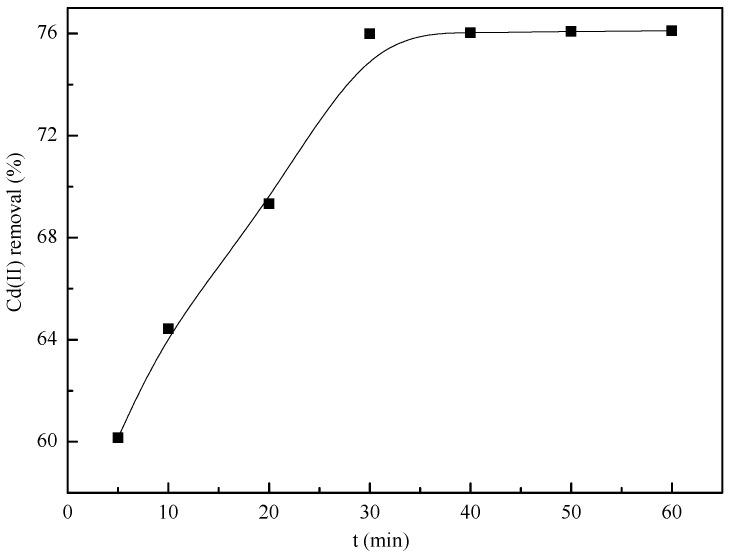
Cd(II) removal kinetics of the nZVI/rGO composites (initial pH = 7.0; nZVI/rGO composites dosage = 30 mg; operating temperature = 20 °C; Cd(II) concentration = 20 mg/L).

Figure 9 shows that the removal process achieves equilibrium within 30 min, and 76.11% of Cd(II) ions is removed by the nZVI/rGO composites with the removal capacity of 25.37 mg/g. The observed increase may be due to the fact that, initially, a large number of vacant sites are available on the adsorbent surfaces for removal and after some time the remaining sites may be difficult to be occupied due to repulsive forces operating between the already adsorbed metal ion on the solid and those present in the solution [68,69].

It is noticed that the calculated *q_e_* value of the pseudo-first-order model is high compared with the experimental *q_e_* value, and the value of R2 is also not high (Table 9). This indicates that the Cd(II) uptake onto the nZVI/rGO composites cannot be approximated favorably by the pseudo-first-order model. However, the calculated *q_e_* value fully agrees with the experimental *q_e_* in the case of the pseudo-second-order model, and the removal data are not well represented by the intraparticle diffusion model with a lower R2 (0.8841).

### 3.8. Cd(II) Removal Mechanisms

The removal of heavy metal ions by the nZVI/rGO composites from aqueous solutions is controlled by various mechanisms, such as reduction, co-precipitation, and adsorption [70,71]. To investigate the interaction mechanisms between Cd(II) and the nZVI/rGO composites, the nZVI/rGO and nZVI/rGO-Cd(II) composites were analyzed by XPS to investigate the elemental composition and valence state on the surface of the nZVI/rGO and nZVI/rGO-Cd(II) composites.

**Figure 10:**
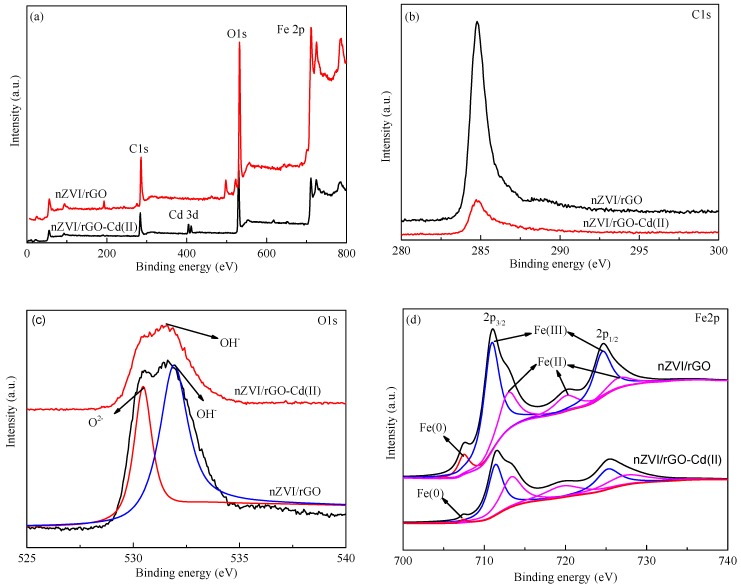

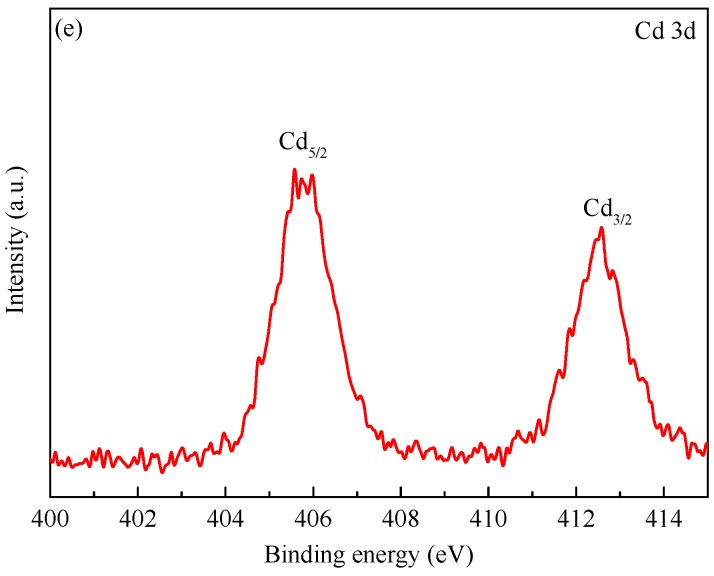
Wide-scan XPS survey of nZVI/rGO and nZVI/rGO-Cd(II) (**a**); high-resolution spectra of C 1s for nZVI/rGO and nZVI/rGO-Cd(II) (**b**); high-resolution spectra of O 1s for nZVI/rGO and nZVI/rGO-Cd(II) (**c**); high-resolution spectra of Fe 2p for nZVI/rGO and nZVI/rGO-Cd(II) (**d**); high-resolution spectrum of Cd 3d for nZVI/rGO-Cd(II) (**e**). (Initial Cd(II) concentration = 100 mg/L; initial pH = 7; nZVI/rGO composites = 300 mg; contact time = 1 h; operating temperature = 20 °C).

The wide-scan XPS spectrum of the nZVI/rGO composites indicated that the surface of the nZVI/rGO composites consists mainly of iron, oxygen, and carbon (Figure 10a). After the treatment by nZVI/rGO composites, Cd(II) appeared in the spectrum of nZVI/rGO-Cd(II) composites. Figure 10b shows the XPS spectra of C 1s for the nZVI/rGO and nZVI/rGO-Cd(II) composites, which demonstrated that nZVI was supported on rGO. The XPS peak of the nZVI/rGO composites did not change before and after the reaction with the Cd(II) ions due to the stability of rGO. As shown in Figure 10c, the O 1s XPS spectrum of the nZVI/rGO composites consist of the two peaks at 530.4 and 531.9 eV, corresponding to the binding energies of O^2−^ and OH^−^, respectively. The O 1s XPS spectrum for the nZVI/rGO-Cd(II) composites has only one peak (531.9 eV), which may indicate that Fe_2_O_3_ disappeared after the reaction with the Cd(II) ions. The high-resolution Fe 2p spectra of the nZVI/rGO and nZVI/rGO-Cd(II) composites show the presence of four iron peaks (Figure 10d). A peak at 706.9 eV corresponding to the binding energy of Fe 2p_3/2_ for the zero-valent iron (Fe^0^) indicates the presence of Fe^0^ on the surface of the nZVI/rGO and nZVI/rGO-Cd(II) composites. This peak for the nZVI/rGO-Cd(II) composites may demonstrate that Fe^0^ did not react with Cd(II). The peaks at 711.2, 720.6, and 724.8 eV are representative of the oxidized iron species (Fe(II) and Fe(III)), which may be present as iron hydroxides (Fe(OH)*_x_*), iron oxyhydroxide (FeOOH), or iron oxides (Fe*_x_*O*_y_*) [72].

The high-resolution Cd 3d spectrum of the nZVI/rGO-Cd(II) composites showed two intense peak for Cd at 405.6 eV for Cd_5/2_ and 412.4 eV for Cd_3/2_ (Figure 10e), which are in accordance with the values reported for Cd(II) in the literature [70]. The results indicate that Cd was removed as the Cd(II) ions on the surface of the nZVI/rGO composites, which may include electrostatic interactions and specific surface bonding. However, our earlier work found that Pb(II) could be reduced by the nZVI/rGO composites [24]. The metal ions with a far more positive standard reduction potential than that of Fe^0^ can be removed by a reduction mechanism, while the metals having a standard reduction potential more negative than or close to that of Fe^0^ can be removed by an adsorption mechanism. The standard reduction potential of Cd(II) (*E_Cd(II)/Cd(_*_0*)*_^0^ = −0.40 V) is close to that of Fe(II) (*E_Fe(II)/Fe(_*_0*)*_^0^ = −0.45 V), while the standard reduction potential of Pb(II) (*E_Pb(II)/Pb(_*_0*)*_^0^ = −0.12 V) is more positive than that of Fe(II). Therefore, the unnoticeable amount of Cd(II) was reduced to Cd(0) by the nZVI/rGO composites, which demonstrated that the removal of Cd(II) by the nZVI/rGO composites could take place by adsorption as reported earlier [19].

## 4. Conclusions

This study investigated the removal of the Cd(II) ions by the nZVI/rGO composites from aqueous solutions by using the RSM and ANN-GA models. The results showed that the ANN-GA model with a higher value of R^2^ and a lower value of average error could provide more accurate predictions than the RSM model in the estimation of process parameters for the Cd(II) removal (initial Cd(II) concentration, initial pH, contact time, and operating temperature). Both ANOVA and sensitivity analysis indicated that initial concentration was the most significant variable for the Cd(II) removal. Using the ANN-GA model, the Cd(II) removal from aqueous solutions was improved by 2.02% in comparison with the RSM model. Therefore, the ANN-GA model may be an ideal alternative for predicting the removal efficiency of Cd(II) by the nZVI/rGO composites, which indicated its potential in predicting the behavior of a complex water treatment process. In addition, the adsorption isotherm and the kinetic model follow the Langmuir isotherm and the pseudo-second-order model, respectively. The result from the Langmuir isotherm illustrated that the Cd(II) removal by the nZVI/rGO composites took place via the monolayer physical adsorption. The calculated thermodynamics parameters exhibit that the removal process was spontaneous and exothermic in nature. The XPS analysis demonstrated that the mechanism for the removal of Cd(II) ions from aqueous solutions was controlled by the adsorption of nZVI/rGO composites. Further studies by using advanced optimization algorithms (particle swarm optimization, Monte Carlo simulation, simulated annealing, etc.) is currently on the way to improving the optimization of pollutant removal from aqueous solutions.

## Figures and Tables

**Table 1:** Independent variables and their levels for RSM modeling and optimization.

Parameters	Unit	Lower Level (−1)	Middle Level (0)	Upper Level (+1)
Initial concentration	mg/L	20	30	40
Initial pH		5	6	7
contact time	min	10	20	30
operating temperature	°C	20	30	40

**Table 2:** Experimental and predicted values of Cd(II) ions removal by nZVI/rGO composites.

Run	*X*_1_	*X*_2_	*X*_3_	*X*_4_	*Y_Experimental_*	*Y_RSM,pred_*	*Y_ANN,pred_*
	mg/L		min	°C	%	%	%
1	40	7	20	30	51.77 ± 0.52	52.48	51.77
2	40	5	20	30	47.05 ± 0.36	46.17	47.05
3	30	5	20	40	50.32 ± 0.42	51.09	50.32
4	20	6	20	40	67.58 ± 0.78	68.35	67.58
5	30	6	20	30	64.88 ± 0.22	64.72	64.74
6	40	6	30	30	50.01 ± 0.57	50.97	50.01
7	30	6	20	30	64.89 ± 0.55	64.72	64.74
8	30	7	20	20	58.97 ± 0.68	58.19	58.97
9	40	6	20	20	44.26 ± 0.15	43.53	44.26
10	30	5	10	30	52.01 ± 0.25	53.01	52.01
11	30	6	30	40	66.97 ± 0.38	65.99	66.97
12	30	6	30	20	58.11 ± 0.21	58.91	58.11
13	20	7	20	30	77.25 ± 0.88	78.09	77.25
14	30	6	20	30	64.62 ± 0.74	64.72	64.74
15	40	6	10	30	49.92 ± 0.28	50.67	49.92
16	20	6	20	20	66.58 ± 0.33	67.43	66.58
17	30	7	20	40	70.85 ± 0.53	71.94	70.85
18	40	6	20	40	56.37 ± 0.45	55.56	56.37
19	30	6	20	30	64.67 ± 0.56	64.72	64.74
20	30	6	20	30	64.52 ± 0.72	64.72	64.74
21	20	6	30	30	75.12 ± 0.52	74.36	75.12
22	30	6	10	40	60.87 ± 0.32	60.04	60.86
23	30	7	30	30	72.88 ± 0.76	71.92	72.88
24	30	7	10	30	61.66 ± 0.11	60.76	64.66
25	20	6	10	30	64.94 ± 0.29	63.98	65.14
26	30	5	20	20	52.98 ± 0.07	51.89	53.79
27	30	5	30	30	51.58 ± 0.26	52.52	55.34
28	30	6	10	20	53.23 ± 0.54	54.18	51.08
29	20	5	20	30	57.99 ± 0.37	57.25	57.28

**Table 3:** ANOVA analysis and statistical parameters for the quadratic model.

Source	Sum of Squares	df	Mean Square	*F*-Value	*p*-Value	Remarks
Model	2109.55	14	150.68	113.96	<0.0001	significant
*X*_1_	1009.80	1	1009.80	763.73	<0.0001	
*X*_2_	552.84	1	552.84	418.13	<0.0001	
*X*_3_	85.55	1	85.55	64.70	<0.0001	
*X*_4_	125.65	1	125.65	95.03	<0.0001	
*X*_1_*X*_2_	52.85	1	52.85	39.97	<0.0001	
*X*_1_*X*_3_	25.45	1	25.45	19.25	0.0006	
*X*_1_*X*_4_	30.86	1	30.86	23.34	0.0003	
*X*_2_*X*_3_	33.93	1	33.93	25.66	0.0002	
*X*_2_*X*_4_	52.85	1	52.85	39.97	<0.0001	
*X*_3_*X*_4_	0.37	1	0.37	0.28	0.6041	
*X*_1_^2^	54.15	1	54.15	40.95	<0.0001	
*X*_2_^2^	71.95	1	71.95	54.42	<0.0001	
*X*_3_^2^	21.76	1	21.76	16.46	0.0012	
*X*_4_^2^	62.66	1	62.66	47.39	<0.0001	
Residual	18.51	14	1.32			
Lack of Fit	18.51	10	1.84	68.85	0.0005	not significant
Pure Error	0.11	4	0.027			
Total	2128.07	28				

**Table 4:** Results from variable importance analysis in the ANN model.

Independent Variable	Order	Relative Importance (%)
Initial concentration	1	43.33
Initial pH	2	30.71
Contact time	4	10.57
Operating temperature	3	15.39

**Table 5:** Comparative results of confirmatory experiments for model validation.

Process Parameters	RSM		ANN-GA	
	Optimized Values	Experimental Values	Optimized Values	Experimental Values
Initial Cd(II) concentration (mg/L)	20.00	20.00	20.16	20.00
Initial pH	7.00	7.00	6.48	6.50
Contact time (min)	30.00	30.00	30.00	30.00
Operating temperature (°C)	37.13	37.10	25.31	25.30
Removal efficiency (%)	85.92	80.36 ± 0.46%	81.50	82.38 ± 0.82%
Average values of prediction errors (%)	6.47		1.08	
R^2^	0.9913		0.9999	

**Table 6:** Parameters of isotherm models for the adsorption of Cd(II) onto the nZVI/rGO composites.

Isotherms	Equation	Parameters	Values of Parameters
Langmuir	$\frac{C_{e}}{q_{e}}=\frac{1}{q_{m}k}+\frac{C_{e}}{q_{m}}$	*k* (L/mg)	0.96
		*q_m_* (mg/g)	47.84
		R^2^	0.9909
Freundlich	$\log q_{e}=\log K_{f}+\frac{1}{n}\log C_{e}$	*K_f_* (mg/g)	20.02
		n 1/n	3.54 0.28
		R^2^	0.9852
D–R	$\ln q_{e}=\ln q_{m}-\beta \varepsilon^{2}$	*β* (mol^2^/J^2^)	10^−7^
		*q_m_* (mol/g) *E* (kJ/mol)	0.34 2.24
		R^2^	0.8226

**Table 7:** The values of R_L_ for the adsorption of Cd(II) ions by the nZVI/rGO composites.

Initial Concentration (mg/L)	*R_L_* Value
5	0.172
10	0.094
20	0.050
30	0.034
40	0.025
50	0.020

**Table 8:** Thermodynamic parameters for Cd(II) removal by the nZVI/rGO composites.

T (K)	Equation	Δ*S* (kJ/mol/K)	Δ*H* (kJ/mol)	Δ*G* (kJ/mol)
293	$\ln k_{c}=\frac{\Delta S}{R}-\frac{\Delta H}{RT}$	0.0587	−8.5759	−25.7752
303				−26.3622
313	ΔG=ΔH−TΔS			−26.9492
323				−27.5363

**Table 9:** Kinetic parameters obtained from pseudo-first-order, pseudo-second-order, and intraparticle diffusion model for the removal of Cd(II) by the nZVI/rGO composites.

Model	Equation	Parameters	Value of Parameters
Pseudo-first-order kinetics	$\log(q_{e}-q_{t})=\log q_{e}-k_{1}t/2.303$	*k*_1_ (1/min)	2.23 × 10^−1^
		*q_e_* (mg/g)	53.43
		R^2^	0.8729
Pseudo-second-order kinetics	$t/q_{t}=1/k_{2}{q^{2}}_{e}+t/q_{e}$	*k*_2_ (g/mg/min)	3.41 × 10^−1^
		*q_e_* (mg/g)	26.32
		R^2^	0.996
Intraparticle diffusion	$q_{t}=k_{3}t^{1/2}+b$	*k*_3_ (mg/g/min^1/2^)	1.03
		*b* (mg/g)	18.37
		R^2^	0.8841
		experimental *q_e_* (mg/g)	25.37

## Supplementary Materials

The following are available online at www.mdpi.com/1996-1944/10/5/544/s1.
