# Supplementary material for: Artificial Neural Network Modeling and Genetic Algorithm Optimization for Cadmium Removal from Aqueous Solutions by Reduced Graphene Oxide-Supported Nanoscale Zero-Valent Iron (nZVI/rGO) Composites
Source: Materials (Basel). 2017 May 17;10(5):544. doi: 10.3390/ma10050544 (PMC5459019; doi:10.3390/ma10050544)
Supplement: Supplementary file 1 [file materials-10-00544-s001.pdf]

Supplementary Information

Artificial neural network modeling and genetic algorithm optimization for cadmium removal from aqueous solutions by reduced graphene oxide-supported nanoscale zero-valent iron (nZVI/rGO) composites

Mingyi Fan [1], Tongjun Li [1], Jiwei Hu [1,*], Rensheng Cao [1], Xionghui Wei [2], Xuedan Shi [1] and Wenqian Ruan [1]

[1] Guizhou Provincial Key Laboratory for Information Systems of Mountainous Areas and Protection of Ecological Environment, Guizhou Normal University, Guiyang 550001, Guizhou, China

[2] Department of Applied Chemistry, College of Chemistry and Molecular Engineering, Peking University, Beijing 100871, China

* Correspondence: jiweihu@yahoo.com; jwhu@gznu.edu.cn; Tel.: +86-851-8670-2710

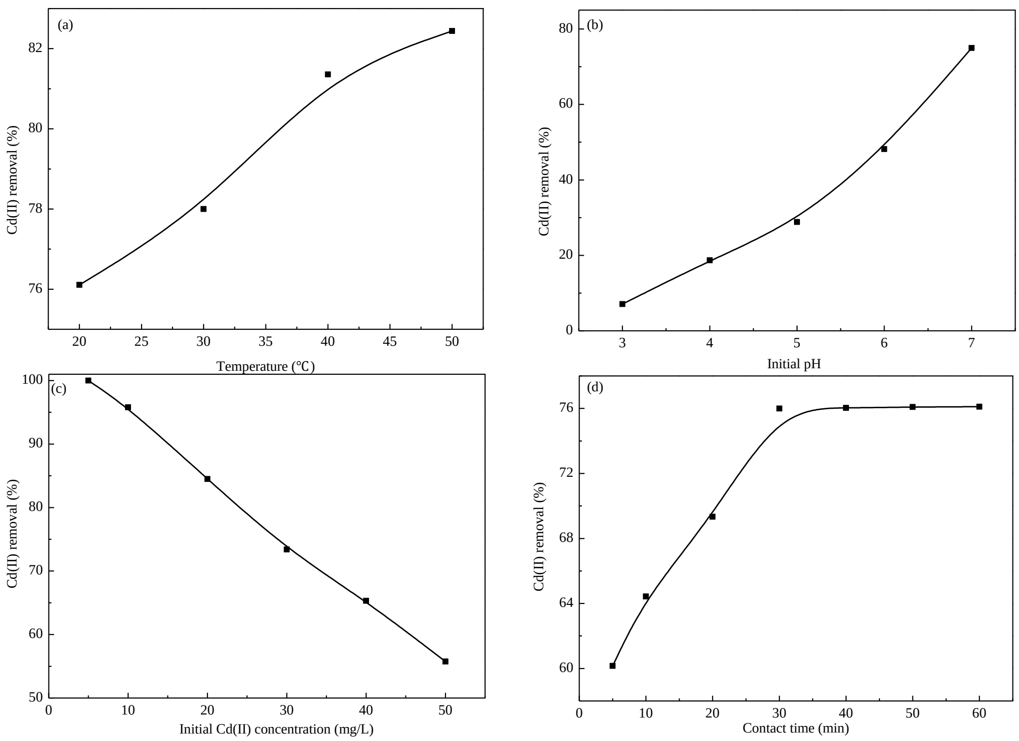

Figure S1. Effect of temperature on Cd(II) removal by nZVI/rGO composites: initial pH = 7.0; nZVI/rGO composites dosage = 30 mg; Cd(II) concentration = 20 mg/L; and time = 1h (a). Effect of initial pH on Cd(II) removal by nZVI/rGO composites: temperature = 20 °C; nZVI/rGO composites dose = 30 mg; Cd(II) concentration = 20 mg/L; and time = 1h (b). Effect of initial Cd(II) concentration removal by nZVI/rGO composites: temperature = 20 °C; initial pH =7.0; nZVI/rGO composites dose = 30 mg; and time = 1h (c). Effect of contact time on Cd(II) removal by nZVI/rGO composites: temperature = 20 °C; initial pH = 7.0; nZVI/rGO composites dose = 30 mg; and Cd(II) concentration = 20 mg/L (d).
